# Contrast-enhanced mammography: what the radiologist needs to know

**DOI:** 10.1259/bjro.20210034

**Published:** 2021-08-31

**Authors:** Lidewij M.F.H. Neeter, H.P.J. (Frank) Raat, Rodrigo Alcantara, Quirien Robbe, Marjolein L. Smidt, Joachim E. Wildberger, Marc B.I. Lobbes

**Affiliations:** ^1^ GROW School for Oncology and Developmental Biology, Maastricht University, Maastricht, the Netherlands; ^2^ Department of Radiology and Nuclear Medicine, Maastricht University Medical Center, Maastricht, the Netherlands; ^3^ Department of Medical Imaging, Laurentius Hospital, Roermond, the Netherlands; ^4^ Department of Radiology, Hospital del Mar, Barcelona, Spain; ^5^ Department of Surgery, Maastricht University Medical Center, Maastricht, the Netherlands; ^6^ Department of Medical Imaging, Zuyderland Medical Center, Sittard-Geleen, the Netherlands

## Abstract

Contrast-enhanced mammography (CEM) is a combination of standard mammography and iodinated contrast material administration. During the last decade, CEM has found its place in breast imaging protocols: after i.v. administration of iodinated contrast material, low-energy and high-energy images are retrieved in one acquisition using a dual-energy technique, and a recombined image is constructed enabling visualisation of areas of contrast uptake.

The increased incorporation of CEM into everyday clinical practice is reflected in the installation of dedicated equipment worldwide, the (commercial) availability of systems from different vendors, the number of CEM examinations performed, and the number of scientific articles published on the subject. It follows that ever more radiologists will be confronted with this technique, and thus be required to keep up to date with the latest developments in the field. Most importantly, radiologists must have sufficient knowledge on how to interpret CEM images and be acquainted with common artefacts and pitfalls.

This comprehensive review provides a practical overview of CEM technique, including CEM-guided biopsy; reading, interpretation and structured reporting of CEM images, including the accompanying learning curve, CEM artefacts and interpretation pitfalls; indications for CEM; disadvantages of CEM; and future developments.

## Introduction

To date, full-field digital mammography (FFDM) remains the primary imaging tool in breast cancer imaging worldwide. FFDM plays a pivotal role in breast cancer detection in clinical practice as well as in screening programmes.^1^ However, FFDM is less accurate in females with dense breast tissue.^2,3^ To resolve this issue, many technologies have been proposed as adjuncts to FFDM, such as digital breast tomosynthesis (DBT), breast ultrasound (US), and breast magnetic resonance imaging (MRI). Contrast-enhanced mammography (CEM) – a combination of mammography and iodinated contrast material administration – is the latest addition, and has consistently been shown to increase diagnostic accuracy as compared to FFDM.^4–6^ Unsurprisingly therefore, CEM is steadily gaining ground, as is reflected in the increasing numbers of CEM equipment, examinations, and published studies.^7^ First commercially introduced in 2011, CEM is now being offered on five different systems by four vendors.^8,9^ Even although system characteristics differ, all available systems use a similar approach and will therefore be uniformly referred to as CEM throughout this review.

A consequence of the growing popularity of CEM is that more and more radiologists will be confronted with this technique. Radiologists will be required to keep up to date with the latest developments in this field and to acquire sufficient knowledge of CEM image interpretation. Most importantly, radiologists need to become acquainted with artefacts commonly seen in CEM and consequent interpretation pitfalls.

The current comprehensive review gives a practical overview and recommendations for CEM technique, including CEM-guided biopsy; reading, interpretation and structured reporting of CEM images, including the accompanying learning curve and an overview of CEM-specific artefacts and interpretation pitfalls; indications for CEM; disadvantages of CEM; and future developments.

### CEM technique: Principles, image acquisition and patient handling

Small tumours depend on diffusion to acquire oxygen and nutrients for their growth. As the tumour expands, diffusion becomes insufficient. Parts of the tumour then become hypoxic, stimulating the release of vascular growth factors. The latter promote new blood vessel formation, ultimately creating vascularization of the tumour itself and providing access to the oxygen and nutrients required for further growth.^10^ These rapidly formed new blood vessels are often ‘leaky’ to contrast agents. As a consequence, after intravascular administration some contrast agent will enter and ‘enhance’ the tumour interstitium. This can be exploited for diagnostic purposes, provided the proper imaging tool is used.^10^ In CEM, iodinated contrast agents are used, usually at a concentration of 300–370 mg iodine/ml.^7^

Intravascular iodinated contrast administration will extend the room time of a typical CEM examination to 15–20 min, which is approximately twice the time required for a FFDM.^11–13^ Contrast agent is administered through an i.v. catheter, usually placed in an antecubital vein, preferably using an automatic injector at rate 2–3 ml s^−1^ and followed by a saline flush at the same flow rate. Before injecting the contrast agent, patency of vascular access is checked by a saline test bolus. Contrast dose is usually 1.5 mL/kg body weight, with a limit on maximum contrast volume (120 cc 300 mg iodine/mL at our institution). Contrast is preferably administered with an automatic injector at rate 2–3 ml s^−1^, followed by a saline flush. Two minutes after contrast injection, the patient is positioned for mammographic imaging. It is recommended to preserve the intravenous access until 15 min after contrast administration, so as to enable prompt treatment of any late adverse reactions to the contrast injection.

It is not necessary to acquire mammographic images in a specific order. Optimally, image acquisition should take place between 2 and 10 min after contrast administration, as all studies have confirmed adequate diagnostic accuracy within this time window. Fortunately, this is more than sufficient for acquiring the standard four mammography views as well as any supplemental views that may be called for. In both FFDM and CEM, exposure time depends on breast size and settings used and generally varies between 4 and 10 s/view.^14,15^ Each CEM view consists of one low-energy (LE) and one high-energy (HE) image, the additional exposure time is in the order of seconds per acquisition,^14^ and breast compression is released in between image acquisitions.

A standard CEM examination consists of a craniocaudal (CC) and a mediolateral oblique (MLO) view of each breast, with supplemental views (such as spot compression view or rolled views) as requested by the radiologist. Vendors have developed varying strategies for dual-energy mammography, using different anode materials, filter materials, and image reconstruction algorithms for combining LE and HE images. A detailed overview of vendor system characteristics has recently been published by Jochelson and Lobbes^9^; an updated overview is given in Table 1.

**Table 1.** System characteristics of the five commercially available CEM systems Updated, from Jochelson and Lobbes^9^.

	GE Healthcare Senographe Essential and Senobright	GE Healthcare Pristina and Senobright HD	Hologic Selenia Dimensions and 3Dimensoins I-View	Siemens Healthineers Mammomat Revelation Titanium CEM	Fujifilm Amulet Innovality CEDM
Low-energy acquisition					
Anode and filter material	Mo & Mo; Mo & Rh; Rh & Rh	Mo & Mo; Rh & Ag	W & Rh; W & Ag	W & Rh	W & Rh
Filter thickness (mm)	Mo, 0.03; Rh, 0.025	Mo, 0.03; Ag, 0.03	0.050	0.050	0.050
Tube voltage range (kV)	26–31	26–34	25–33	28–34	26–31
High-energy acquisition					
Anode and filter material	Mo & Al + Cu; Rh & Al + Cu	Mo & Cu; Rh & Cu	W & Cu	W & Ti	W & Al + Cu
Filter thickness (mm)	Al, 0.3; Cu, 0.3	0.25	0.3	1.0	Al, 0.7; Cu, 0.25
Tube voltage range (kV)	45–49	49	45–49	49	45–49
Complete CEM examination					
Mean glandular dose (mGy)	1.6–2.8	0.7–2.3	3.0	1.7	1.4
Total acquisition time (sec)	3–8	2–5	<2	15–22	5

Ag, silver; Al, aluminum; CEDM, contrast-enhanced digital mammography; Cu, copper; Mo, molybdenum; Rh, rhodium; Ti, titanium; W, tungsten.

**Figure 1.**
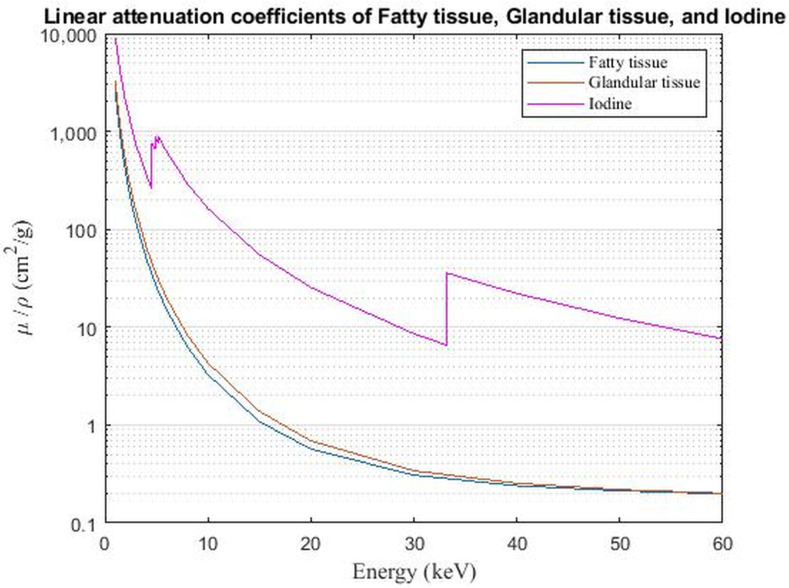
Principle of iodine-based contrast enhancement. Mass attenuation coefficients of fatty tissue, glandular tissue, and iodine are shown. The iodine curve shows a steep elevation in attenuation at 33.2 keV, which is the k-edge of iodine. Differences in attenuation between breast tissue and iodinated contrast material are larger beyond the k-edge of iodine. Thus in high energy images (44–49 kVp), the differences in attenuation are larger than in low-energy images (26–30 kVp). Image processing of low- and high-energy images subsequently results in recombined images, showing contrast enhancement overlay.

CEM makes use of the photoelectric effect of iodine which enables highlighting areas of contrast uptake. The photoelectric effect itself depends on the energy of the X-ray beam and k-edge of the material. The absorption k-edge of iodine (33 keV) falls within the average range of the X-ray beam in mammography. Furthermore, iodine X-ray absorption, or mass attenuation coefficient, is higher than that of breast tissue (Figure 1).

During image acquisition, first the LE image is acquired using tube voltages varying between 26 and 30 kVp.^15–19^ Even although iodinated contrast is already present within the breast at this point, the LE mean energy falls below the k-edge of iodine and, as several studies show, LE is equivalent to FFDM in terms of image quality.^20–22^

The HE image is acquired second. In HE image-acquisition, the X-ray beam ranges from 44 to 49 keV. A photoelectric effect occurs when an incoming 44–49 keV photon causes an electron from the k-shell of an iodine atom to eject, thereby increasing the attenuation of iodine. Because iodine contrast has ‘leaked’ into the tumour interstitium, the latter will be enhanced and the difference between tumour and breast tissue becomes more apparent.^23^

**Figure 2.**
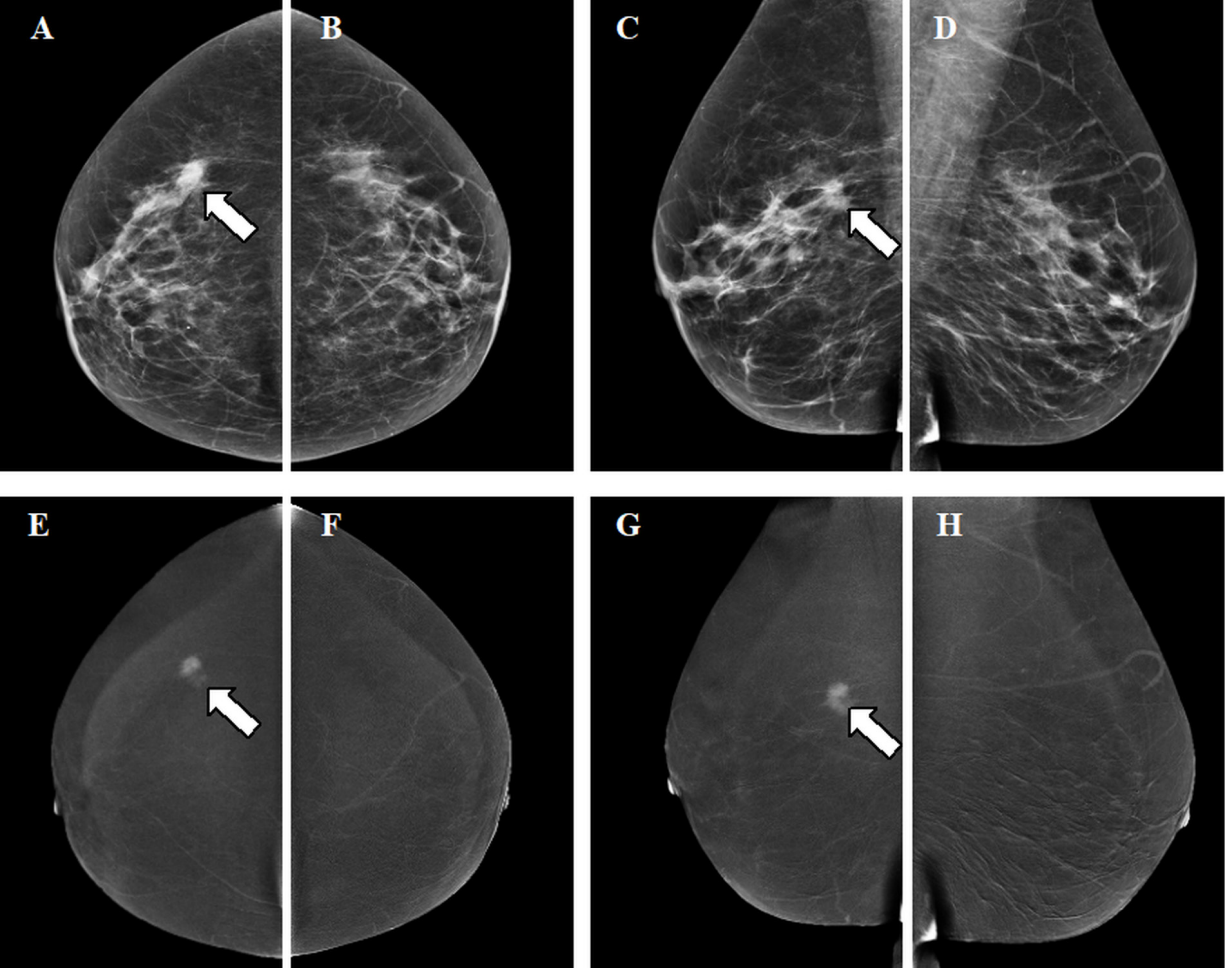
Contrast-enhanced mammographic images in a 67-year-old female recalled from the breast cancer screening program because of a new, spiculated mass in the right breast. A-D. Low energy images. E-H. Recombined images. Images were acquired of the right and left breast in craniocaudal (CC) and mediolateral oblique (MLO) views. The mass in the right breast is visible on low-energy images in both CC and MLO views (arrows in A and C). The recombined images of the right breast show enhancement of the lesion in both CC and MLO views (arrows in E and G). Histopathological results showed an invasive breast cancer of no special type, Grade 2, size 1.4 cm.

**Figure 3.**
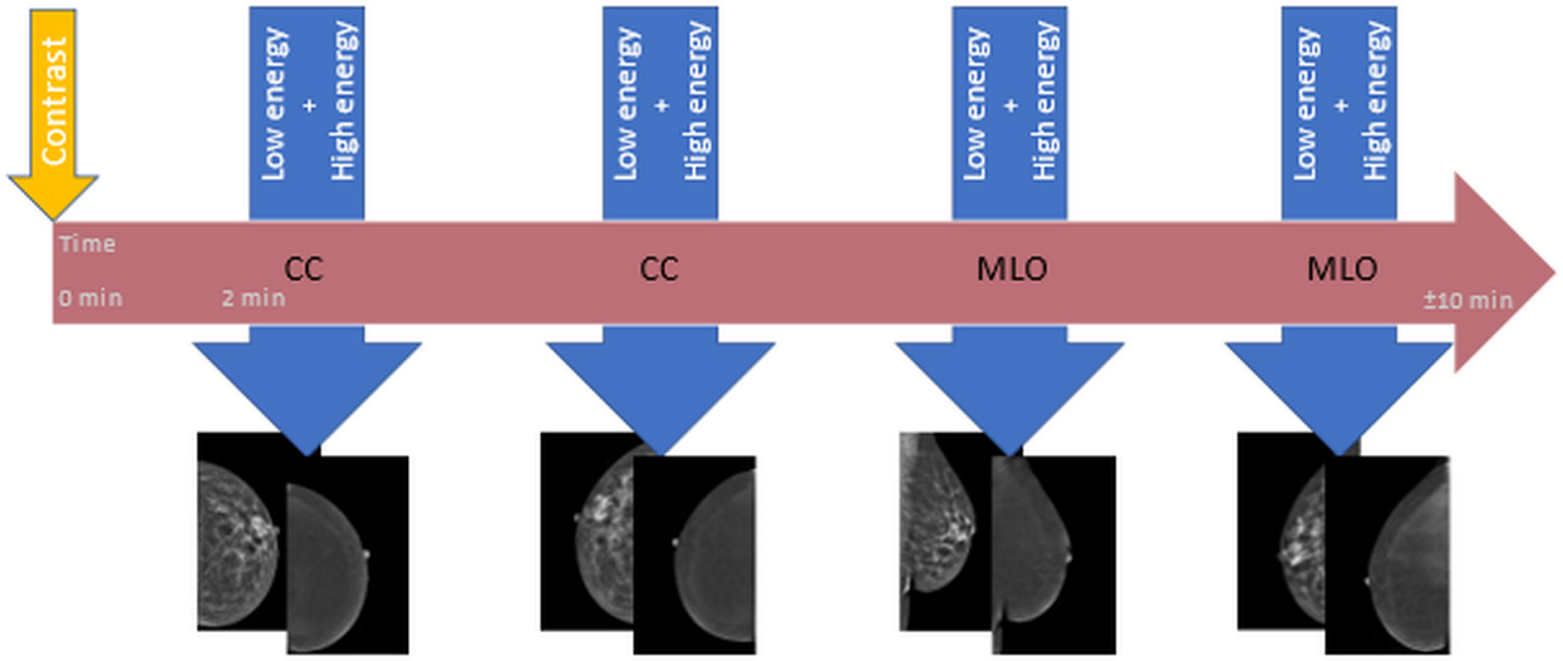
Diagram of image acquisition for contrast-enhanced mammography. The horizontal arrow represents the time window of 10 min in which a full (at least four views) contrast-enhanced mammography examination must be performed in order to be considered of diagnostic value. The iodine-based contrast agent is administered at time point zero (small vertical arrow), 2 min prior to the acquisition of the first view. Per view, one low energy and one high energy image are acquired within one compression (larger vertical arrows). The order of views may differ. After image processing, low energy and recombined images are retrieved for clinical assessment.

Although the HE image contains relevant information, this cannot be perceived by the human eye. The information is instead used in post-processing to construct the so-called recombined or iodine image showing areas of contrast uptake. The end-result of the imaging process is LE and recombined CEM images from both breasts in two standard views (see example in Figure 2; an overview of a standard image-acquisition protocol is presented in Figure 3).

### Reading, interpretation and reporting CEM images

#### CEM learning curve

CEM is easy to learn, especially when readers have some experience with FFDM and MRI. This is supported by the results of the multi-reader study by Lalji et al,^24^ in which seven radiologists and three residents assessed 199 cases (first LE images, followed by the complete CEM examination). Three levels of experience were distinguished: residents with marginal experience in CEM/FFDM; radiologists with at least two years’ experience in CEM/FFDM; and radiologists with extensive experience in FFDM but none in CEM. Specificity and diagnostic performance increased significantly with CEM compared to FFDM regardless of level of experience. CEM sensitivity scores achieved by the residents (96.6%) and non-experienced CEM readers (95.9%) were similar to those of experienced readers (97.6%). These results suggest that novice CEM readers can reach a level equal to that of experienced radiologists.^24^ This is supported by another study in which non-experienced high-school students, after a short introduction to breast cancer and CEM in general, evaluated the cases used in the study by Lalji et al. These students immediately reached a sensitivity of more than 80% in detecting breast cancers on recombined images.^25^ This also implies that semi-automatic software tools that are being developed might show steep learning curves (see ‘Future developments’).

It is not easy to determine how many CEM examinations must be read in order to be considered an experienced reader.^26^ To the best of our knowledge, the only available study covering this specific topic is the one by Cheung et al, showing that a radiologist should read an average of 75 CEM examinations to reach a 90% probability of correct prediction.^27^ Based on the above observations and the wide availability of CEM examinations, it is safe to assume that a minimum of 75 cases should be practised to acquire sufficient experience in clinical practice.

#### Hanging protocol

In practice, LE images are interpreted first to assess morphologic abnormalities, the recombined image being used for extra information.^24,27^ This is the ‘standard’ hanging protocol proposed by the different vendors. However, alternative hanging protocols are feasible. To illustrate this, Van Geel et al compared CEM diagnostic accuracy using the ‘standard’ hanging protocol and an inverse hanging protocol (*i.e.,* first interpret the recombined image, followed by the LE image).^28^ They found that sensitivity and specificity were equivalent between standard and inverse protocols, 98 and 99 versus  94% and 90%, respectively, but that the inverse hanging protocol led to an average decrease in reading time of 6.2 s/case. This was mainly due to shorter LE image evaluation in the inverse hanging protocol, average recombined image evaluation time remained similar.^28^ Although time differences are small, they may become of interest in situations where large volumes of CEM examinations must be read, as is the case in screening programmes.

#### CEM artefacts

CEM can show artefacts, either related to the LE image or specific to the technique itself. Artefacts seen on the LE image are similar to those observed in FFDM and include air trapping, antiperspirant on the skin mimicking (micro)calcifications, and disruption of the X-ray beam by matter such as hair.^29,30^ In general, these artefacts are well known and can be easily resolved by repeating image acquisition.

**Figure 4.**
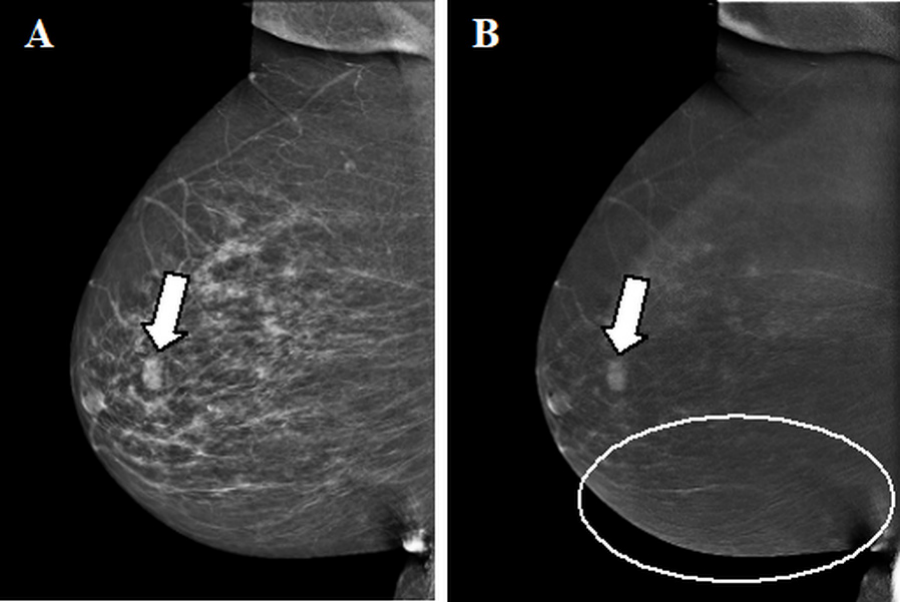
Enhancing fibroadenoma. A,B. Contrast-enhanced mammographic of right breast in mediolateral oblique view in a 63-year-old female recalled from screening because of a new ill-defined and partly obscured mass. A. Low-energy image showing the suspect mass (arrow in A). B. Corresponding recombined image in which the suspect lesion is showing enhancement (arrow in B). The lines visible in the caudal part of the breast (circle) are the result of slight motion between the low- and high-energy image acquisition, the ripple artefact. Histopathological results showed a classic fibroadenoma.

**Figure 5.**
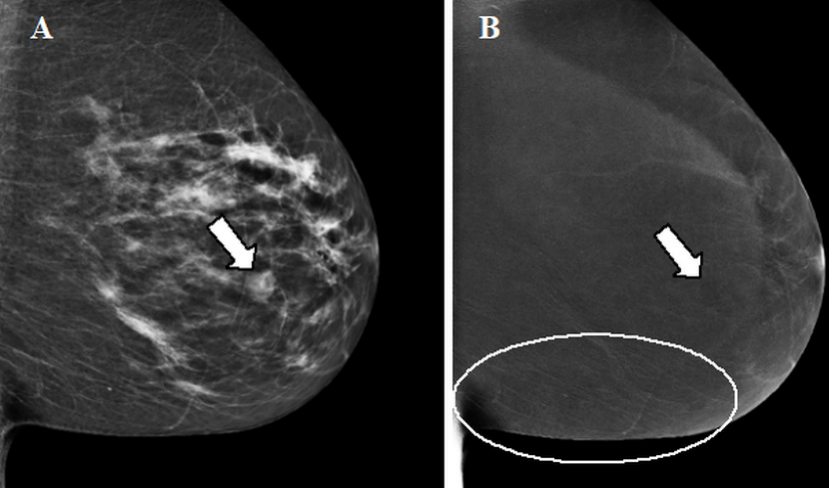
Contrast-enhanced mammographic images in a 55-year-old female recalled from screening because of a new mass in the left breast. A. Low-energy image in mediolateral oblique view shows an ill-defined round mass (arrow in A). B. Corresponding recombined image. At the site of the suspect lesion a subtle ‘eclipse sign’ is visible, implicating a cyst (arrow in B). No screen-detected interval breast cancer has been reported in the 18-month follow-up period. The ripple artefact is also visible on the recombined image (circle).

**Figure 6.**
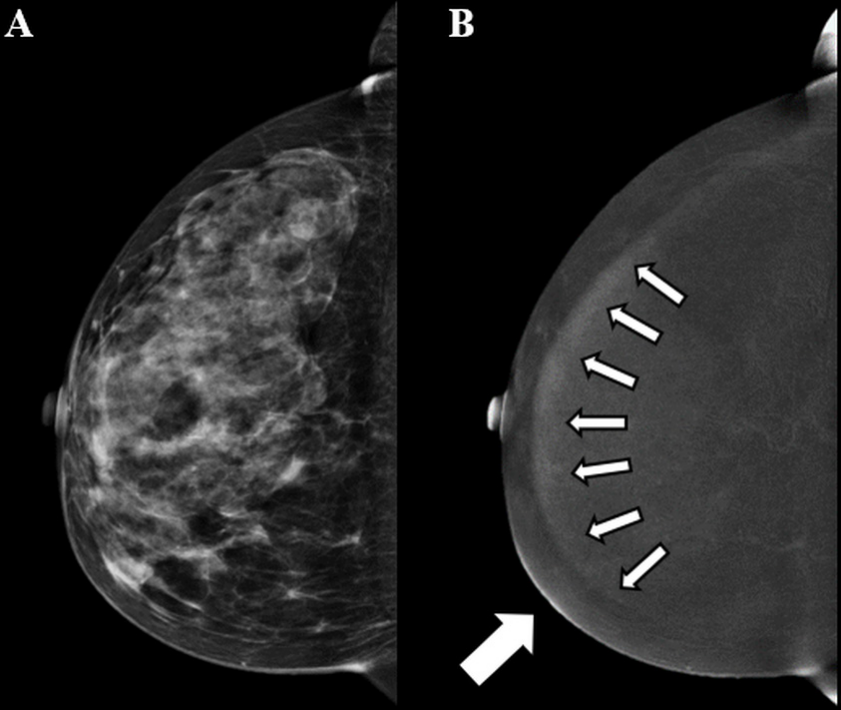
Contrast-enhanced mammographic images in craniocaudal view in a 63-year-old female. A. Low-energy image of the right breast. B. The rim artefact is shown in the recombined image (small arrows). In addition, the skin line enhancement artefact is visible in the anteromedial part of the breast (larger arrow). No suspicious findings were reported.

Some artefacts are specific to CEM and visible on the recombined image. An overview of these artefacts, their causes, and potential solutions, is provided in Table 2 (for artefact illustrations see Figures 4–6).^9,29–37^

**Table 2.** Overview of CEM specific artefacts

Artefacts	Cause	Appearance on recombined image	Solution
Ripple artefact	Slight motion of the breast between the LE and HE image acquisition. More often seen in increasing breast thickness and mainly in MLO view.	Thin black and white lines in a ripple-like structure (see circles in Figures 4 and 5).	Reduce movement of the patient during acquisition through patient instruction.
Rim artefact or “breast-in-breast”	Scattered radiation. In older systems: misalignment of the LE and HE anti scatter grids.	Double-breast contour in the form of a brighter breast edge-shape mimicking a “breast-in-breast” (see small arrows in Figure 6). This can be visible in both CC and MLO views.	Most commonly observed on CEM exams performed on first generation systems, less or not applicable in newer systems.
Skin line enhancement	Image filtration to equalize breast thickness.	The skin contour is partially highlighted (see larger arrow in Figure 6).	Most commonly observed on CEM exams performed on first generation systems, less or not applicable in newer systems. Can be easily dismissed if absence of any skin-related problems on the LE image.
Breast implants	Image distortion.	Poor recombined image quality with black or white areas surrounding implant.	Use other imaging modalities, such as MRI.
Axillary line artefact	Wrong usage of the small compression paddle.	Horizontal lines in the axillary region on the recombined image.	Use correct paddle size for large breasts.
Contrast splatter	Contamination of contrast on the skin (not a true technical artefact).	May mimic small (micro)calcifications on the LE image.	Prevent contamination by wearing gloves during contrast administration and/or washing hands before patient positioning. Distinction is easy: in contrast splatter, the corresponding lesion is extremely bright, whereas calcifications are black on the recombined image.
Skin lesions	Skin lesions such as haemangiomas showing enhancement, superimposed within the boundaries of the breast.	Mimics an intramammary enhancing lesion.	Check for noticeable skin lesions which might correspond to an enhancing lesion during breast positioning.

CC, cranio-caudal; CEM, contrast-enhanced mammography; HE, high-energy; LE, low-energy; MLO, mediolateral oblique; MRI, magnetic resonance imaging.

#### Interpretation pitfalls

It is important to note that some lesions, such as invasive lobular carcinomas and mucinous carcinomas, are more difficult to detect using CEM. Van Nijnatten et al showed that invasive lobular carcinomas often show weak enhancement. On LE images such lesions appear as architectural distortions or asymmetries (instead of masses), rendering them difficult to spot on either type of CEM image.^38^ Mucinous carcinomas contain large amounts of fluid and only limited numbers of vital tumour cells, and thus have limited blood supply (perfusion). As such, they only enhance slightly, or show rim enhancement, or sometimes show no enhancement at all.^24^ Hence, the absence of enhancement in morphologically suspicious lesions cannot rule out breast cancer, and the CEM recombined image must therefore be seen as an adjunct to mammography rather than a replacement. Besides these two tumour types, cancers can be inherently difficult to detect due to their location in the mammographic field-of-view. CEM being a mammographic technique, some lesions may be overlooked in mammography blind spots, such as the medial part of the breast, the inframammary fold, the prepectoral zone, and the axillary tail.^24,39^ Lesions in these areas are difficult to visualize in both FFDM and CEM, despite optimal breast positioning. If lesions are (partially) observed or suspected in these areas, breast MRI should be considered.

On the other hand, benign lesions can show enhancement on CEM, potentially resulting in false-positive findings. Common benign causes of enhancement are: fibroadenomas (Figure 4), atypical ductal hyperplasia, papilloma, infection or inflammation and radial scars.^24,40,41^ Of the 128 benign lesions examined by Tsigginou et al, 37 showed enhancement on CEM (28.9%).^40^ A similar percentage of enhanced benign lesions was seen by Deng et al. (12/44), and results suggest that the probability of a malignancy increases with stronger enhancement.^42^ Although false-positive findings may lead to unnecessary biopsies or follow-up examinations, studies have shown that they occur less frequently in CEM than in FFDM.

#### Structural reporting of CEM examinations

LE images, being equivalent to FFDM, can be interpreted using the terminology suggested in the latest edition of the ACR BI-RADS lexicon.^43–45^ To some extent, recombined images are comparable to standard MRI examinations, and therefore the use of standard MRI terminology is recommended when describing enhancement of lesions. For example, masses may be homogeneously or heterogeneously enhanced, or may show (irregular) rim enhancement. If no masses are observed, but instead architectural distortion or asymmetry is seen, the term ‘non-mass enhancement’ can be used in CEM reports, and the different characteristics described accordingly. However, some artefacts are specific to CEM and have acquired specific descriptions. For example, negative enhancement with or without a thin rim of enhancement also known as an ‘eclipse sign’, is the specific appearance of a cyst on CEM (Figure 5).^40,46,47^ In addition, there are artefacts specific to recombined CEM images.

The amount of background parenchymal enhancement (BPE) in CEM can also be described as minimal, mild, moderate or marked enhancement, using terminology similar to that of MRI.^48,49^ An increase in BPE is associated with increased odds for breast cancer.^48,49^ The majority of patients showed to have minimal-to-mild BPE on CEM.^48,49^ In a study by Sogani et al, three experienced breast imaging readers compared BPE levels between CEM and MRI showing agreements on BPE levels varying from moderate to substantial with κ = 0.55; κ = 0.66, and κ = 0.67.^49^ Hence, interference of BPE is more or less comparable between the assessment of CEM and MRI.

At present, CEM is being considered for the ACR BI-RADS lexicon, and a comprehensive overview of structural reporting in CEM is expected to be available soon. Until that time, the recommendation is to keep LE and recombined image findings separate in the report, matching them where necessary, and to base the final BI-RADS classification on the complete CEM examination.^27^

#### Indications for CEM

The three most common indications for CEM are inconclusive findings, pre-operative staging, and response monitoring. Evidence of CEM efficacy in these settings, however, is mainly based on retrospective studies,^7^ and proposed indications should be considered with this in mind. Current prospective trials such as the RACER and CMIST are ongoing and will provide scientific evidence for these indications.^50,51^

#### Inconclusive findings

One of the most studied aspects of CEM is its ability to act as ‘problem solving’ tool in the setting of inconclusive findings in conventional imaging, foremost a recall from the breast cancer screening programme. Despite low disease prevalence, CEM was shown to increase sensitivity, specificity, positive-predictive value (PPV), and negative-predictive value (NPV) in this population.^24,46^

A feasibility study by Zuley et al suggests that CEM significantly reduces the false-positive rate (FPR) (*p* = 0.017) and significantly increases the true-positive rate (TPR) (*p* = 0.019) in BI-RADS 4 soft tissue lesions compared to FFDM/DBT.^52^ Even in combination with ultrasound, the TPR of FFDM/DBT did not match that of CEM whilst the FPR significantly increased. Based on these results, CEM is likely to be more accurate than a FFDM/DBT/US combination. Moreover, supplemental US after negative CEM findings is questionable: the risk of finding false-positive lesions is increased without any real improvement in terms of cancer detection.^52^

The benefit of CEM in assessing suspicious breast calcifications is not as clear. A prospective study by Cheung et al in patients with screening recalls for suspicious microcalcifications found 88.9% sensitivity and 86.6% specificity.^53^ In a similar study, Houben et al found a slight increase in diagnostic accuracy, with only 81.1% of ductal carcinoma *in situ* (DCIS) showing enhancement, but it was not sufficient to be of added value for clinical use in surgical treatment planning.^54^ Considering these findings, it is currently not recommended to downgrade unenhanced calcifications to a lower BI-RADS classification. On the other hand, enhancement of calcifications may be sufficient grounds for an upgrade of the BI-RADS classification, but biopsy remains necessary.

For patients with contraindications for MRI (claustrophobia, pacemaker, metallic implant), CEM is a good alternative; diagnostic performance appears to be comparable.^55–57^ In a recent review by Xiang et al, pooled sensitivity was found to be 97% for both CEM and MRI, whereas accuracy and pooled specificity were higher for CEM: 98 and 66 versus  92% and 52%, respectively.^57^ These pooled results may not be applicable to specific study populations. In a prospective study with BI-RADS 3–5 lesions comparing diagnostic performance of multiple breast imaging modalities including CEM, the best diagnostic performance was achieved using MRI.^58^ Nevertheless, CEM performance makes it quite an acceptable alternative to breast MRI when the latter is not preferred or unavailable. However, relative strengths and weaknesses of each modality need to be investigated in more detail: in specific subpopulations and for diagnostic accuracy certainly, but also regarding cost-efficiency.

#### Pre-operative staging

Breast MRI is currently the reference standard for assessing tumour extent and presence of additional foci.^59^ CEM has been evaluated as a tool for pre-operative staging and may provide a good alternative for MRI. CEM tends to slightly overestimate tumour size (in the order of mms’), while FFDM/LE and ultrasound tend to underestimate tumour size, compared to histological size.^55,60–62^ Size measurements using CEM are comparable to those using MRI, and both are in concordance with or slightly overestimated compared with histological size.^58,59,61,63–65^

A single-centre retrospective study in the setting of preoperative breast staging (*n* = 326) found 93% sensitivity and 98% specificity for CEM. Furthermore, CEM led to a change in surgery type compared to conventional imaging in 18.4% of patients.^66^ It is mostly symptomatic patients with palpable lesions who benefit from staging with CEM. In a study with 101 CEM-detected lesions, CEM led to 17 additional imaging and 12 additional biopsies, and the surgical treatment plan was changed for 20 patients.^39^

#### Response monitoring

Neoadjuvant chemotherapy (NAC) is increasingly used to treat locally advanced breast carcinomas. The aim of NAC is to reduce tumour size, thereby decreasing the need for mastectomy and/or lymph node dissection. In response monitoring, the tumour is usually assessed before, during and after treatment. Response to NAC is reflected in a decrease in tumour size as well as in changes in lesion enhancement. Before CEM, MRI was the most accurate imaging modality for tumour extent measurements and residual tumour evaluation.^67–69^ However, initial results of studies on CEM in response monitoring are encouraging.

In a study by Iotti et al, 46 patients underwent both MRI and CEM before, during and after treatment. CEM better predicted the pathological complete response than MRI (Lin’s coefficient 0.81 and 0.59, respectively); both imaging modalities underestimated the size of residual tumours, 4.1 mm on average for CEM and 7.5 mm on average for MRI.^59^ In a similar study among 33 patients by Barra et al, Lin’s coefficients of 0.7 and 0.4 were found, and residual tumour size was overestimated with an average of 8.0 mm for CEM and 18.0 mm for MRI.^70^ Both studies suggest CEM to be more accurate than MRI in residual tumour evaluation.^59,70^ A first systematic review and meta-analysis of CEM and MRI in response monitoring was recently published, including 6 CEM and 21 MRI studies. Pooled sensitivity for CEM was higher than that of MRI, 83vs 77%, whereas pooled specificities were equal, 82vs 82%.^71^ Available data are limited, but so far CEM appears to be a good alternative to MRI in response monitoring.

#### CEM-guided biopsy

**Figure 7.**
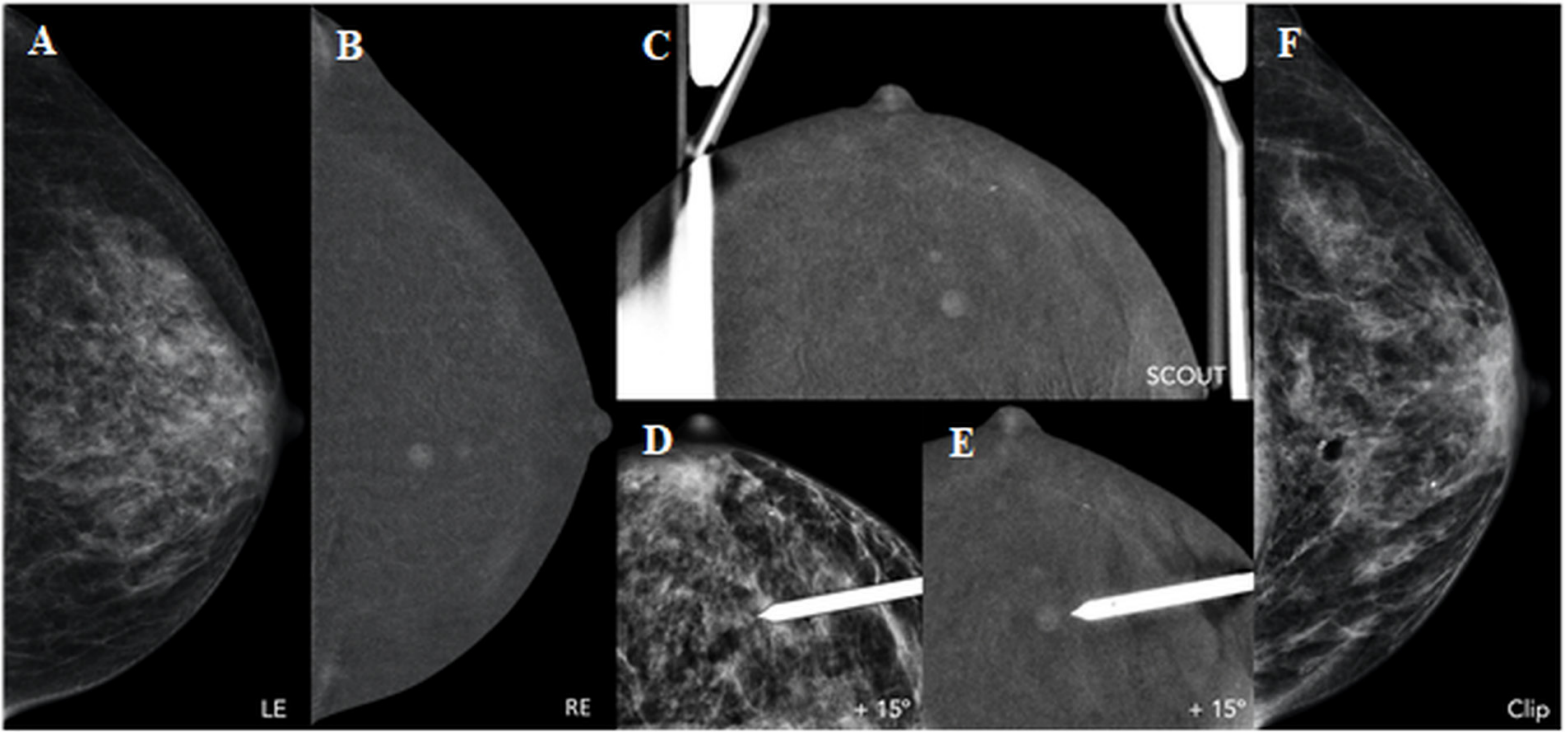
CEM-guided biopsy in a 61-year-old patient with palpable lesion in right breast (IDC, not shown) and additional contralateral (left breast) finding on diagnostic CEM. A,B. Low-energy (LE) and recombined image (RE) of left breast in craniocaudal view. There is a 6 mm mass enhancement at 12 o’clock, with no ultrasound correlation and not enough references on 2D/3D in order to favour a conventional mammographic-guided biopsy. C-E. The procedure of CEM-guided biopsy is similar to a standard stereotactic biopsy (one scout and a pair of angled stereotactic images) with the additional step of contrast media injection 2 min before compression and first imaging. Like a routine CEM, each acquisition is composed of one low-energy (LE) and one high-energy (HE) exposure. The inclusion of the enhancing lesion is confirmed with a recombined scout view (0°), followed by the two angled views. Another pair of stereotactic angled images (** ± **15°) is sometimes acquired, previous to fire-forward, in order to confirm that the target was reached. F. Final CC view after clip placement. Histopathological results showed an invasive lobular carcinoma in the left breast.

CEM-guided biopsy was developed to access enhancing lesions not seen on accompanying LE images or targeted US. It is a promising alternative to MRI-guided biopsy. The technique may be used to guide various interventional procedures of the breast, such as vacuum-assisted biopsy or excision (VAB or VAE), core needle biopsy, and pre-surgical wire localization. CEM-guided biopsy is based on the principle of (conventional) stereotactic procedures, using dual energy acquisition and i.v. administration of iodinated contrast media. Image acquisition is performed in a similar way to diagnostic CEM, including the 2-min wait after contrast administration (Figure 7). Inclusion of the enhancing lesion is confirmed with a recombined scout view (0°), after which a pair of dual-energy angled stereotactic images ( ± 15°) is acquired with the objective indicated in each. Thus, the equipment automatically calculates the X, Y and Z coordinates allowing access to the target. Generally, enhancement will be visible for at least 5 to 7 min which is sufficient for target selection. After local anaesthesia, a needle is inserted into the breast until the limit point is reached, as defined by the support. Another pair of stereotactic angled images is sometimes acquired before the fire-forward to confirm that the objective was reached, or to redefine coordinates if it was not. Next, sampling is carried out with the vacuum system device. We recommend to extract at least 12 tissue samples in order to reduce sampling error. Lastly, it is crucial to mark the biopsy bed with a radiological marker, ideally using the same probe.

#### Disadvantages of CEM

CEM has two important disadvantages: the use of iodinated contrast agents and an increase in radiation dose. Potential benefits of CEM should always be weighed against these disadvantages.

#### Iodinated contrast material

Although the use of modern iodine-based contrast materials is considered safe, a possibility of mild, moderate or severe anaphylactoid reactions remains.^72^ In a systematic review, Zanardo et al found the pooled rate of adverse reactions in CEM examinations to be 0.82%.^7^ At our institution, we observed a 0.6% rate of adverse reactions in CEM examinations.^41^ However, subjects with prior hypersensitivity reactions to any of the ingredients of iodinated contrast should be excluded from undergoing CEM, since breast MRI could be considered a safer alternative.

In addition to hypersensitivity reactions, iodinated contrast administration may cause post-contrast acute kidney injury. Patients at risk of acute kidney injury, such as those with renal insufficiency, incur a risk when undergoing CEM.^73^ Since breast imaging never involves ‘do or die’ scenarios, alternative methods for diagnostics such as breast MRI should be used in such cases, in accordance with safety guidelines on the use of iodinated contrast material.^73,74^

#### Radiation dose

The first study on CEM radiation dose performed on a commercially available system approved by the U.S. Food and Drug Administration (FDA) (as opposed to a prototype or modified unit) was performed by Badr et al. They found a 54% higher mean radiation dose for CEM (2.65 mGy) than for FFDM (1.72 mGy).^75^ Three other studies comparing CEM and FFDM radiation dose similarly found higher doses for CEM.^14,76,77^

Studies thus consistently find an increase in radiation dose for CEM, but the magnitude differs. This is presumably due to variation in system settings and different patient characteristics, projection views and breast thickness for example, may influence results. An overview of the various study characteristics is presented in Table 3.^14,75–77^

**Table 3.** Overview of studies comparing radiation dose of CEM to FFDM on FDA approved, commercially available systems

	CEM					FFDM					Difference
Article	Patients (n)	Images (n) and VIEWS (n/n)	Mean breast thickness (mm) + (range)	Mean AGD (mGy) + (range)	System (vendor)	Patients (n)	Images (n) and VIEWS (n/n)	Mean breast thickness (mm) + (range)	Mean AGD (mGy) + (range)	System (vendor)	Dose ratio CEM / FFDM (%)
Badr *et al*. (2014)	104	391 CC/MLO (N/A)	56 (N/A)	2.65 (N/A)	Not reported	104	360 CC/MLO (N/A)	57 (N/A)	1.72 (N/A)	Not reported	54%
Jeukens *et al*. (2014)	47	195 CC/MLO (96/97)	58 (21-96)	2.80 (1.10–4.29)	Senographe Essential +SenoBright (GE Healthcare)	715	2782 CC/MLO (1238/1339)	56 (15-100)	1.55 (0.63–5.12)	Senographe Essential +SenoBright (GE Healthcare)	81%
James *et al* (2017)	173	174 single CC	63 (N/A)	3.0 (N/A)	Selenia Dimensions (Hologic)	6214	6215 single CC	47 (N/A)	1.8 (N/A	Selenia Dimensions (Hologic)	42%
Phillips *et al*. (2018)	45	180 CC/MLO (90/90)	56 (22-88)	2.49 (N/A)	Senographe Essential +SenoBright (GE Healthcare)	45	180 CC/MLO (90/90)	56 (N/A)	1.40 (N/A) 2.16 (N/A)	Senographe Essential (GE Healthcare) Selenia Dimensions (Hologic)	78% 15%

AGD, average glandular dose; CC, cranio-caudal; CEM, contrast-enhanced mammography; FFDM, full-field digital mammography; MLO, mediolateral oblique.

Although increased, CEM radiation dose remains within safe radiation dose limits according to the Mammography Quality Standards Act regulations (3.0 mGy per view).^78^ The life-attributable risk (LAR) number for cancer incidence incurred by a complete CEM exam with four acquisitions at the age of 40 is 0.009% , and the LAR for cancer mortality is even lower, at 0.002%. These percentages drastically decrease with increasing age.^41,79^ Nevertheless, the As Low As Reasonably Achievable (ALARA) principle is also applicable to CEM, meaning that risks should always be weighed against benefits.

#### Future developments

Continual technical developments are being explored to further advance CEM. These not only include technical hardware improvements but also advances in the post-processing algorithm, which may help to reduce CEM artefacts and improve image quality in general.

Enhancement plays an important role in the evaluation of CEM examinations, and there seems to be diagnostic information encompassed in the amount of enhancement than can be observed. For example, Lobbes et al found that grey values on recombined images were significantly higher for malignant lesions than for benign lesions (*p* = 0.002) or cysts (*p* < 0.001).^80^ Unfortunately, such differences cannot be accurately assessed through visual inspection and grey values of enhancement are difficult to quantify. Herein lies an opportunity for the use of artificial intelligence and radiomics.

Indeed, machine-learning algorithms with textural and morphological features are already able to distinguish benign lesions from malignancies with an accuracy of 90% (45/50).^81^ Moreover, initial results from Marino et al reveal radiomics accuracies of 78 to 100% in differentiating between malignant lesions based on several tumour characteristics, such as (non-)invasiveness, three hormone receptor sensitivities (positive or negative), and tumour grade (Grades 1–3).^82^ Finally, Wang et al created a radiomics monogram using 14 radiomics features and risk factors, and achieved an accuracy of 81% in predicting tumour response to NAC using CEM.^83^ Currently, ongoing studies use deep learning algorithms to detect breast lesions on CEM and radiomics to subsequently classify them. The introduction of machine learning-based decision support tools for CEM appears to be only a matter of time.

## Conclusion

Since its commercial introduction in 2011, CEM has been steadily incorporated as imaging tool in clinical practice. CEM is surprisingly easy to learn and confers logistic and diagnostic advantages over breast MRI. However, it is a relatively novel addition and future studies will certainly elaborate on its strengths and weaknesses, not only in terms of specific populations and diagnostic accuracy, but also in cost-effectiveness.
